# Optineurin overexpression ameliorates neurodegeneration through regulating neuroinflammation and mitochondrial quality in a murine model of amyotrophic lateral sclerosis

**DOI:** 10.3389/fnagi.2025.1522073

**Published:** 2025-02-07

**Authors:** Shumin Zhao, Ranran Chen, Yi An, Yali Zhang, Cheng Ma, Ying Gao, Yanchao Lu, Fei Yang, Xue Bai, Jingjing Zhang

**Affiliations:** ^1^Department of Neurology, Chifeng Municipal Hospital, Chifeng, China; ^2^Chifeng Clinical Medical College of Inner Mongolia Medical University, Chifeng, China; ^3^Medical Research Center, Chifeng Municipal Hospital, Chifeng, China; ^4^Intensive Care Unit, Chifeng Municipal Hospital, Chifeng, China

**Keywords:** amyotrophic lateral sclerosis, apoptosis, mitochondrial quality, neuroinflammation, optineurin

## Abstract

**Introduction:**

Amyotrophic lateral sclerosis (ALS) is a neurodegenerative disease characterized by the loss of motor neurons (MNs). Genetic mutations in Optineurin (OPTN) and Superoxide Dismutase 1 (SOD1) have been identified as causal factors for ALS. OPTN immunopositive inclusions have been confirmed in the cases of ALS with SOD1 mutations. However, the role of the OPTN gene in ALS caused by SOD1 mutations is ambiguous.

**Methods:**

The murine Optn lentivirus and empty vector lentivirus were injected into SOD1^*G*93*A*^ mice after discovering variations in Optn expression over time. The phenotype onset date, life span, locomotor activity, and pathological changes in the spinal cord were determined and recorded subsequently. In addition, the influences on cellular apoptosis, mitochondrial dynamics, mitophagy, and neuroinflammation were further investigated.

**Results:**

Optn expression was increased in the spinal cord of SOD1^*G*93*A*^ mice at the pre-symptomatic phase, but decreased after disease onset. Optn overexpression led to a 9.7% delay in the onset of disease and improved motor performance in SOD1^*G*93*A*^ mice. Optn overexpression also ameliorated the MNs loss by 46.8%. Moreover, all these ameliorating effects induced by Optn overexpression might be due to the inhibition of cellular apoptosis, improvement of mitochondrial quality, regulation of mitochondrial dynamics, promotion of mitophagy, and anti-inflammatory properties.

**Conclusion:**

Our data demonstrate that Optn overexpression protects MNs, inhibites cellular apoptosis, improves mitochondrial quality and regulates neuroinflamation in SOD1^*G*93*A*^ mice at the pre-symptomatic stage.

## 1 Introduction

Amyotrophic lateral sclerosis (ALS) is a progressive and fatal neurodegenerative disease characterized by the degeneration of upper and lower motor neurons (MNs) in the cerebral cortex, brainstem, and spinal cord, leading to muscle atrophy and paralysis. Most ALS patients die from respiratory failure within 2–5 years of disease onset (Feldman et al., 2022). ALS can be categorized as either familial or sporadic based on its inherited genes, with 10% of cases having a family history of ALS, known as familial ALS (fALS). The remaining cases without affected family members are classified as sporadic ALS (sALS). In China, approximately 25% of familial ALS (fALS) cases are attributed to mutations in the superoxide dismutase 1 (SOD1) gene. Although SOD1 mutations occur in only 1–2% of sporadic ALS (sALS) cases, SOD1 remains the most common causative gene in our sALS population (Wei et al., 2017). Several hypotheses have been proposed regarding the pathogenesis of ALS, such as mitochondrial dysfunction, autophagy defect, glutamate excitotoxicity, and abnormal RNA metabolism. Despite these various hypotheses, the exact mechanism underlying the disease remains incompletely understood. Consequently, very limited effective therapeutics have been approved by FDA for current ALS treatment (Jaiswal, 2019).

Optineurin (OPTN) gene mutation is also one of the causes of fALS, OPTN gene encodes the OPTN cytoplasmic protein, which consists of several structural domains, including zinc finger domain, ubiquitin-binding domain, LC3 binding domain, and coil-coiled domain (Maruyama et al., 2010). It plays a variety of physiological roles including mitophagy, protein aggregation, neuroinflammation, and vesicle trafficking in the central nervous system (CNS), where it is widely expressed (Minegishi et al., 2016). OPTN protein can also interact with proteins such as myosin VI, Rab8, Huntingtin, and TANK-binding protein 1, which are involved in several cellular processes including membrane transport, cytokinesis, vesicular transport, transcriptional activation, and actin-microtubule reorganization (Sundaramoorthy et al., 2015).

It has been reported that the distinct OPTN immunoreactivity of ubiquitin and TDP-43-positive intracytoplasmic inclusions on serial sections from patients with sALS (Maruyama et al., 2010). Moreover, the distinct OPTN immunopositive lewy-body-like hyaline inclusions were also confirmed in the cases of ALS caused by chromosome 9 open reading frame 72 (C9orf72), fused in sarcoma (FUS) and SOD1 mutations (Hortobágyi et al., 2011). OPTN gene may play a fundamental role in the pathophysiology of ALS, and its function deserves further research (Zhao et al., 2023). One study found that mutant SOD1 protein sequester Optn into aggregates, which interferes with the mitophagy process in N2a neuroblastoma cells (Tak et al., 2020). Recently, Wen and colleagues demonstrated that Optn mediates its therapeutic effects by regulating mitophagy in SOD1^*G*93*A*^ mice and cells (Wen et al., 2024). However, Optn dynamics had not been studied in the spinal cord of SOD1^*G*93*A*^ mice before and after disease onset, and neuroinflammation had not been investigated in SOD1^*G*93*A*^ mice after Optn overexpression. Here, we investigated Optn dynamics in the lumbar spinal cord of SOD1^*G*93*A*^ mice at both pre-symptomatic and symptomatic stages. Furthermore, we also examined the effects of Optn overexpression in vivo by injecting lentivirus into the lateral ventricular of SOD1^*G*93*A*^ mice. The possible molecular mechanisms such as neuroinflammation, apoptosis and mitochondrial quality were further explored.

## 2 Materials and methods

### 2.1 Plasmid construction and lentiviral encapsulation

The mouse Optn (NM_001356487.1) coding sequencewas amplified by polymerase chain reaction (PCR). The full-length cDNA of the Optn gene was cloned into the pLVX-mOPTN-mCMV-ZsGreen lentiviral vector and sequenced for confirmation. The cloned vector and packaging components were co-infected with lentivirus-encapsulated HEK293T cells. The supernatant was collected by centrifugation after 48 h and concentrated using a concentration tube for backup. This technology was provided by Likely Biotechnology, Beijing, as shown in Supplementary Figure 2A.

### 2.2 Mice and treatments

Transgenic SOD1^*G*93*A*^ mice expressing mutant human SOD1 with a Gly93Ala substitution (B6SJL-Tg-SOD1^*G*93*A*^-1Gur, No.002726) mice were kindly donated by Xiaojie Zhang of Shanghai Sixth People’s Hospital. The mice were bred in the SPF animal laboratory of Chifeng Municipal Hospital. Pure congenic versions of this mouse were nonviable, while heterozygotes exhibited pathological changes and symptoms similar to ALS. Male transgenic (Tg) mice were mated with female wild-type (Wt) B6SJL mice of the same genetic background in order to maintain and breed the obtained Tg animals.

At 30 days of age, the mice were identified using PCR to amplify mouse tail DNA and determine the mouse genotypes according to our previous report (Zhang et al., 2018), it was used to determine whether the offspring were Wt or Tg mice. Then, male mice in asymptomatic and symptomatic stages at 40 days and 90 days of age were chosen, randomizing into four groups: 40-day-old Wt mice (Wt40d), 40-day-old Tg mice (Tg40d), 90-day-old Wt mice (Wt90d), and 90-day-old Tg mice (Tg90d) (3 mice per group). The main objective of present study was to investigate alterations in Optn protein expression within the spinal cord of these mice across different stages of the disease progression.

Optn overexpression in the symptomatic phase: An Optn overexpression group (Tg-Optn) and a control group (Tg-NC) were established by randomly selecting and assigning eighteen symptomatic male SOD1^*G*93*A*^ mice at 90 days of age, with 9 mice in each group. The Tg-Optn group received a single-injection of 10 μl of Optn overexpression lentiviral vector (titer 5 × 10^8^/mL) into the right lateral ventricle (AP: –0.22 mm, ML: –1.0 mm, DV: –2.35 mm), while the Tg-NC group was injected with an equal volume of empty lentivirus (Chen et al., 2015). The Rotarod Test was utilized to assess the locomotor ability of the mice, alongside recording their weight changes and survival times.

Optn overexpression in the pre-symptomatic phase: 50 male Tg mice were randomly divided into Tg-Optn and Tg-NC groups of 25 mice each. Mice in the Tg-Optn group were single-injected with 10 μl of Optn-overexpressing lentiviral vector (titer 5 × 10^8^/mL) into the right lateral ventricle at 60 days of age. Mice in the Tg-NC group were injected with an equal amount of lentiviral vector (Chen et al., 2015). A total of 12 mice from each group were monitored for disease onset and survival duration, with their locomotor abilities assessed using the rotarod test, onset dates determined, and weight changes and survival times recorded. The remaining 13 mice from each group were sacrificed at 90 days of age, and samples from their lumbar segments were extracted for pathological, molecular analysis and transmission electron microscopy analysis.

The animal experiments involved were approved by the Experimental Animal Ethics Committee of Chifeng Municipal Hospital and were conducted in accordance with the National Institutes of Health guidelines for the care and use of laboratory animals.

### 2.3 Assessment of body weight, motor function and lifespan

The body weight of each mouse was measured and recorded once every 2 days, began from the lentiviral injection until death, in order to compare the weight changes between the two groups.

For motor function assessment, the rotarod test was conducted after 7 days of training, maintaining a constant speed of 20 rpm from the age of 70 days. This test was performed once every 2 days. The mice were allowed to run on the rotarod apparatus for a maximum of 5 min and the latency to fall off the rotating rod was recorded. The onset time of disease was defined when animals fell off the rotarod three times within 5 min (Zhang et al., 2014).

As per animal ethics guidelines, the determination of the mouse’s death date involved assessing their hind limb mobility and ability to consume food. When a mouse displayed complete paralysis of the hind limbs and inability to eat, it was positioned on a flat surface with its back toward the ground. Failure to autonomously return to its normal position within 30 s marked the official time of death, prompting the required euthanasia process (Koh et al., 2007).

### 2.4 Lumbar spinal cord extraction

Eight mice per group were used to extract the lumbar spinal cord. At 90 days of age, mice were anaesthetized by injection intraperitoneally with 1.25% tribromoethanol (0.2 mL/10 g body weight) and perfused transcardially with 40 mL of pre-cooled phosphate-buffered saline (PBS), then four lumbar spinal cords were extracted and stored at −80°C refrigerator for further protein cleavage. The remaining four mice continued to be perfused with 20 mL of 4% paraformaldehyde (PFA) in a fast and then slow manner. The spinal cord of lumbar segments was extracted on ice, immersed in 4% PFA, and fixed overnight. Then, tissues were immersed in 15 and 30% sucrose solutions for 24 h each to dehydrate and precipitate the sugar, respectively. Finally, the tissues were embedded using OCT and rapidly stored at −80°C refrigerator. OCT-embedded tissues were placed in a −20°C frozen sectioning machine (Leica) for serial sectioning at a thickness of 10 μm and mounted on electrostatically coated slides.

### 2.5 MNs counting

The spinal cord frozen sections were initially placed into a pre-prepared 1% toluidine blue staining solution for 10 min. They were then removed, rinsed using distilled water, and consecutively immersed in gradient alcohol dehydration solutions of 70, 80, 90, and 100% for 2 min each. Subsequently, sections were washed with xylene solution twice (5 min each time), then sealed by neutral gum and dried naturally. Finally, the sections were observed and photographed using the microscope (Olympus BX51). The unilateral anterior horn of each animal was examined by a technician who was unaware of the experimental design. A total of 20 sections, 10 μm thick, were collected at 10-slice intervals from each animal. To count MNs, each of the 4 sections of serial sections (50 per mouse) was stained with Nissl staining. The counting of MNs in the unilateral anterior horn of each slice was performed in a blinded manner based on the following criteria: (i) their location in the anterior horn region before the spinal cord’s central canal, (ii) a diameter greater than 20 μm, and (iii) the presence of distinct nuclei (Zhang et al., 2018).

### 2.6 TUNEL staining

TUNEL staining was utilized to specifically detect apoptosis using standard protocol. Briefly, frozen sections were first fixed with 4% PFA for 30 min and then washed three times with PBS for 10 min each. PBS containing 0.5% Triton X-100 was then added to the frozen sections and incubated for 5 min at room temperature. The sections were then washed twice with PBS and incubated in TUNEL staining solution for 60 min at 37°C, followed by three washes with PBS. Finally, the sections were sealed with an anti-fluorescence quenching solution (Solarbio, S2100) and observed under an Olympus fluorescence microscope.

### 2.7 Immunofluorescent staining

Frozen lumbar spinal cord sections were rinsed three times with PBS for 5 min each, then blocked and permeabilized with 5% bovine serum albumin (BSA) in PBS containing 0.3% Triton X-100 for 1 h at room temperature. Slides were then incubated with primary antibodies Optn (1:400, Proteintech, 10837-1-AP), SMI-32(1:1000, Abcam, ab8135), Iba1 (Wako, 1:500, 019-19741), GFAP (Proteintech, 1:200, 16825-1-AP), LC3 (Proteintech, 1:200, 14600-1-AP) and P62 (Proteintech, 1:200, 18420-1-AP) at 4°C overnight. After rinsing with PBS, the sections were incubated for 90 min at room temperature in the dark with the appropriate secondary antibodies Alexa Fluor 592 (Proteintech, 1:2,000) and Alexa Fluor 488 (Cell Signaling, 1:2,000). The sections were then washed with PBS and sealed with an anti-fluorescence quenching sealer. They were then observed under the Olympus fluorescence microscope. In the ventral part of the spinal cord removed from each mouse, the number of Ibal-positive microglia and GFAP-positive astrocytes with intact cytoplasm was counted, statistical analyses were then performed.

### 2.8 Cell counting and relative fluorescence density measurement

To conduct TUNEL staining and immunofluorescence cell counting, five sections per mouse, spaced 100 μm apart, were photographed under a microscope. Each section was imaged bilaterally in the anterior horn region, capturing two fields of view per section. Subsequently, an individual blinded to the study grouping counted the number of positively stained cells in the bilateral anterior horn region. For statistical analysis, the average number of positive cells from the five sections per mouse was calculated, and the number of positive cells per mm^2^ was determined. Additionally, the relative fluorescence density of P62 staining was analyzed using ImageJ software (Schneider et al., 2012).

### 2.9 Immunoblotting

Total tissue protein was extracted from lumbar spinal cord of Tg-NC and Tg-Optn mice with Radio Immunoprecipitation Assay (RIPA) protein lysis buffer containing 1% phenylmethanesulfonylfluoride (PMSF) and protease inhibitor. After determining the protein concentration, 15 μg of protein was denatured at 100°C for 5 min and mixed with sample loading buffer. The denatured samples were then loaded onto sodium dodecyl sulfate-polyacrylamide gels for separation, and subsequently transferred to polyvinylidene fluoride membranes. The membranes were then incubated with primary antibodies Optn (1:2,000, Proteintech, 10837-1-AP), β-actin (1:2,000, Proteintech, 20536-1-AP), GAPDH (1:2,000, Proteintech, 60004-1-Ig), CD206 (1:2,000, Proteintech, 18704-1-AP), CD86 (1:1000, Santa Cruz, sc-28347), OPA1 (1:2,000, Proteintech, 27733-1-AP), Mfn1 (1:2,000, Proteintech, 13798-1-AP), Mfn2 (1:2,000, Proteintech, 12186-1-AP), Drp1(1:2,000, Proteintech, 12957-1-AP), Mff (1:2,000, Proteintech, 17090-1-AP), PINK1 (1:2,000, Proteintech, 23274-1-AP), Parkin (1:1,000, Proteintech, 14060-1-AP), TOM40 (1:1,000, Santa Cruz, sc-365467), TIM23 (1:1,000, Santa Cruz, sc-514463), Cytochrome c (1:2,000, Proteintech, 10993-1-AP), Bcl-2 (1:2,000, Proteintech, 68103-1-Ig) and Bax (1:10,000, Proteintech, 50599-2-Ig) at 4°C overnight, after blocking with 5% non-fat milk powder for 1 h at room temperature and three 10-min washes in tris buffered saline with tween-20 (TBST). The membranes were then incubated with HRP-conjugated goat anti-mouse IgG or goat anti-rabbit IgG secondary antibodies for 1.5 h at room temperature. Finally, the membranes were washed three times and imaged using a BIORAD chemiluminescence imager, using the Enhancement of Luminescence Kit (Proteintech, PK10003) as the substrate for visualization.

### 2.10 Quantitative real-time PCR

Total RNA was extracted from spinal cord samples of Tg-NC and Tg-Optn mice (*n* = 3 in each group) using the TransZol Up (Transgene, ET111-01-V2). Subsequently, The extracted RNA was synthesized to cDNA using the EasyScript^®^ First-Strand cDNA kit (Transgene, AE301-02). For the quantification of gene expression, real-time PCR was performed with the TransStart^®^ Top Green qPCR SuperMix (Transgene, AQ131-01), and the results were monitored by the BIO-RAD real-time PCR system. The primer sequences were provided upon request as summarized in Table 1. The relative expression levels of each primer sequences mRNA were analyzed by the 2^–ΔΔCt^ algorithm normalizing to GAPDH and relative to the Tg-NC groups.

**TABLE 1:** The primer sequences used in the PCR.

	Forward (5′–3′)	Reverse (5′–3′)
GAPDH	CCAATGTGTCCGTCGT GGATCT	GTTGAAGTCGCAGGAG ACAACC
Optn	TGTCCCATCAACCTCTGA GCTGCCT	AGGCAGCTCAGAGGTT GATGGGACA
TNF-α	CAGGCGGTGCCTAT GTCTCA	TCCTCCACTTGGTG GTTTGC
TGF-β	GAGGCGGTGCTCG CTTTGTA	GGGCACTGCTTCCC GAATGT
IL-1β	CTCAACTGTGAAAT GCCACC	GAGTGATACTG CCTGCCTGA
IL-10	GGCAGAGAAGCATGGC CCAGAA	AATCGATGACAGCGC CTCAGCC

### 2.11 Transmission electron microscopy

Fresh tissues were collected from the L4-5 lumbar spinal cord of Tg-NC and Tg-Optn mice within 1–3 min and sized to 1 mm^3^. Samples were then transferred to Eppendorf tubes containing fresh electron microscope fixative and stored at 4°C. The tissues were then washed 3 times for 15 min each with 0.1 M PBS (phosphate-buffered, pH 7.4). The samples were initially fixed with 1% OsO4 in 0.1 M PB (pH 7.4) for 2 h at room temperature, followed by post-fixation in dark. Subsequently, the tissues were rinsed in 0.1 M PB (pH 7.4) three times for 15 min each to eliminate the OsO4. Following this, the tissues underwent sequential dehydration in increasing concentrations of ethanol (30, 50, 70, 80, 95%, and two rounds of 100%) for 20 min each. Finally, 100% acetone was applied twice for 15 min in each round to complete the dehydration process. The tissue samples underwent a series of steps for resin infiltration and embedding. Initially, samples were treated with a mixture of Acetone and EMBed 812 in a 1:1 ratio for 2–4 h at 37°C, followed by another round of treatment with Acetone and EMBed 812 in a 1:2 ratio overnight at 37°C. Subsequently, the samples were subjected to pure EMBed 812 for 5–8 h at 37°C. Once the infiltration process was completed, the pure EMBed 812 was poured into the embedding plates, and the tissues were inserted into the pure EMBed 812, followed by an overnight incubation at 37°C. The embedding plates containing the resin and samples were then transferred to a 65°C oven for polymerization, which lasted for more than 48 h. Subsequently, the resin blocks were removed from the embedding plates and set aside at room temperature for further processing. The resin blocks were then sliced into thin sections of 60–80 nm on an ultramicrotome, and the tissues were carefully transferred onto 150-mesh cuprum grids with formvar film. The samples were subsequently stained with a 2% uranium acetate saturated alcohol solution in the dark for 8 min, followed by rinsing in 70% ethanol three times and then in ultra-pure water three times. Additionally, a 2.6% lead citrate solution was used for staining to avoid CO_2_-induced artifacts, followed by rinsing with ultra-pure water three times. After being dried with filter paper, the cuprum grids were placed in a grid box and left to dry overnight at room temperature. Finally, the cuprum grids were examined under a TEM, and images were captured for analysis. In the electron microscopic analysis, two groups of ALS model mice, Tg-NC and Tg- Optn, were employed, with each group consisting of five mice. From the spinal cord samples of each mouse, fifteen electron microscope images, including those of MNs, were randomly selected for examination. A blinded evaluator, who was unaware of the experimental groupings, conducted the analysis by counting and measuring the number and maximum diameter of mitochondria in each image. The average values derived from these measurements were subsequently used for statistical analysis. All TEM techniques in this experiment were provided by Wuhan Servicebio Technology Co., Ltd.

### 2.12 Statistical analysis

The data were presented as Mean ± SD. Statistical analyses were performed using one-way or two-way ANOVA to a normal distribution. Non-parametric tests were performed for non-normally distributed data by using SPSS 24.0. In addition, the time to onset and survival data were statistically examined using Kaplan-Meier survival analysis. Graphs of the results were generated using GraphPad Prism 8. Statistical significance was set at a *P*< 0.05.

## 3 Results

### 3.1 Optn was significantly increased in the spinal cord of SOD1^G93A^ mice presymptomatically but dramatically decreased after disease onset

**FIGURE 1:**
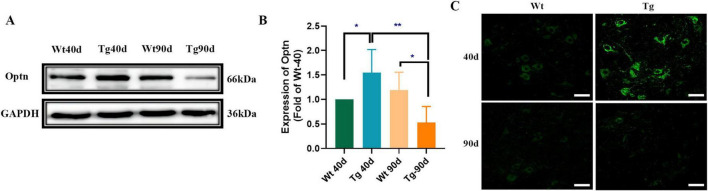
Optn expression changed in the lumbar spinal cord of SOD1^G93A^ mice. **(A)** Optn expression in lumbar spinal cord of Wt and Tg mice, at 40 and 90 days of age by Western Blot. Data are expressed as Mean ± SD. *n* = 3 mice for each condition. (Two-way ANOVA reveals a significant effect, *P < 0.05 and **P < 0.01). **(B)** Relative level of Optn in Wt and Tg mice. **(C)** Immunostaining of Optn in MNs of the two groups of mice at different points time (*P < 0.05; **P < 0.01; scale bar, 50 μm).

Western blotting was used to determine Optn protein expression in the lumbar spinal cords. Our data indicated that the Optn protein in spinal cords of 40-day SOD1^G93A^ mice was approximately 50% higher than that of age-matched Wt mice. However, after the disease onset (at 90 days), Optn expression decreased significantly and was about 60% lower than Wt mice (Figures 1A,B). To identify the type of cells in which Optn is mainly expressed, lumbar spinal cord sections from SOD1^G93A^ mice were immunofluorescently stained with antibodies against Optn. Consistent with the Western blotting results, immunofluorescence staining revealed similar dynamics of Optn expression in the lumbar spinal cords of SOD1^G93A^ mice and Optn predominantly stained on neurons (Figure 1C and Supplementary Figure S1). This aligns with previous studies that demonstrate Optn’s specific localization in neurons and its close association with the pathological processes of various neurodegenerative diseases (Mori et al., 2012; Korac et al., 2013; Nakamura et al., 2014; Wise and Cannon, 2016; Wen et al., 2024). These findings suggest that pathology-related changes in Optn levels may be involved in neurodegeneration during ALS progression.

### 3.2 Optn overexpression in the pre-symptomatic phase delayed the disease onset time and improved motor performance in SOD1^G93A^ mice

In order to determine the effect of the Optn overexpression in the spinal cord, Optn protein levels were assessed by immunoblotting at 1, 2, 3, and 4 weeks after Optn lentivirus injection in Wt mice. Our findings demonstrated a significant increase in Optn expression in the spinal cord after 1, 2, 3, and 4 weeks of injection compared to control group. Moreover, the Optn protein levels remained consistent throughout this 4-week period (Supplementary Figures S2B,C). We also confirmed a significant increase in Optn expression in the spinal cord of SOD1^G93A^ mice after 4 weeks of lentiviral infection by western blot and fluorescence photomicrography (Supplementary Figures S2D–F).

**FIGURE 2:**
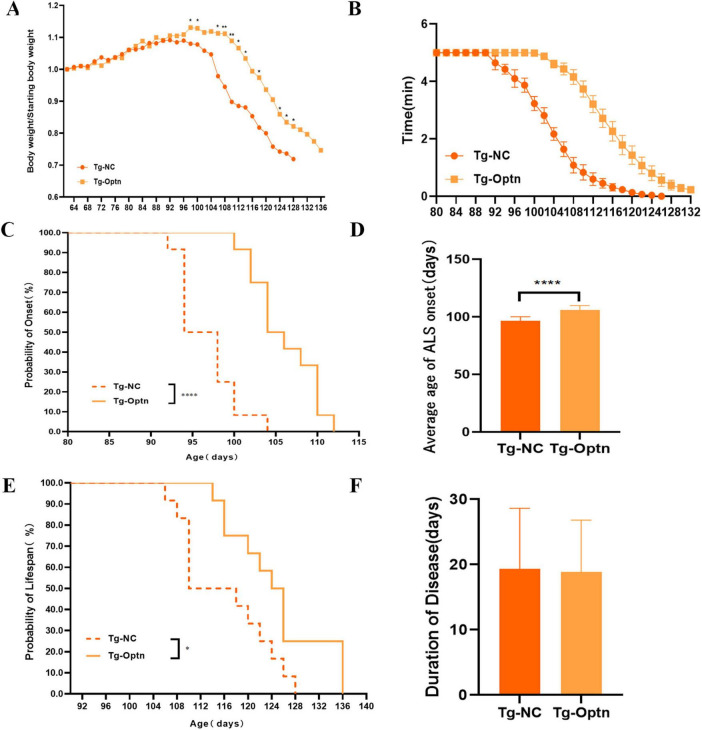
The impact of Optn overexpression on body weight, onset time, survival, and locomotor activity in SOD1^G93A^ mice. **(A)** Body weight curves in the Tg-NC and Tg-Optn mice. **(B)** Rotarod time at constant speeds of 20 rpm. **(C)** Age of ALS onset of Tg-NC and Tg-Optn mice. **(D)** Average days at the ALS onset of Tg-NC and Tg-Optn mice. **(E)** Survival curve of Tg-NC and Tg-Optn mice. **(F)** Duration of Tg-NC and Tg-Optn mice. Data are expressed as Mean ± SD. Compared with the Tg-NC group, *P < 0.05, **P < 0.01, and ****P < 0.0001, *n* = 12 in each group.

Moreover, our findings revealed that the time of weight loss in Tg-Optn mice was notably later than that in Tg-NC mice, as indicated by the lower decrease rate curve for Tg-Optn mice compared to Tg-NC mice (Figure 2A). Furthermore, Tg-Optn mice exhibited a longer mean stick-turning time than Tg-NC mice at the same age (Figure 2B). Additionally, Optn overexpression significantly postponed disease onset by approximately 9.7% (106.00 ± 3.91 days vs. 96.67 ± 3.55 days, *P* < 0.01) at the pre-symptomatic stage (Figures 2C,D) and extended survival by about 7.2% (124.83 ± 7.88 days vs. 116.00 ± 7.82 days, *P* < 0.05) of SOD1^G93A^ transgenic mice (Figure 2E), but failed to extend the duration of the disease (Figure 2F).

Before that, we also increased the expression of Optn in symptomatic (90-day-old) SOD1^G93A^ mice. However, subsequent statistical analysis revealed that Optn overexpression had no significant effect on delaying the time of disease onset, reducing weight loss or prolonging survival at 90 days of age (Supplementary Figures S3A–D). These results strongly indicate that Optn maybe beneficial in improving ALS condition before the disease onset.

### 3.3 Optn overexpression alleviated MNs apoptosis in the spinal cord of SOD1^G93A^ mice

**FIGURE 3:**
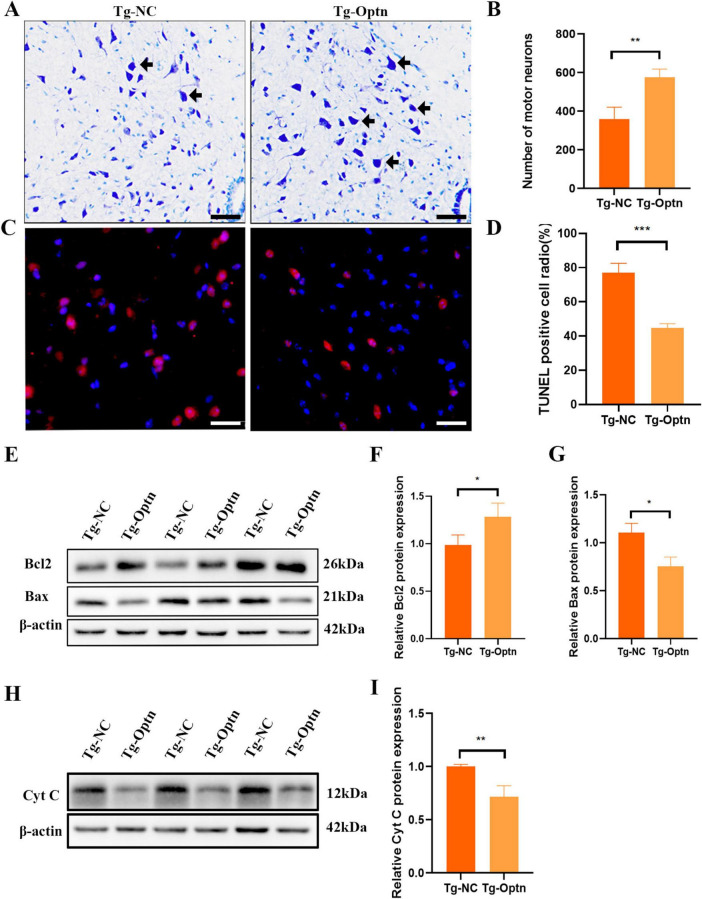
Optn overexpression reduced MNs loss and inhibited apoptosis in ALS mice. **(A)** Representative photomicrographs of Nissl-staining in lumbar spinal cord of the Tg-Optn and Tg-NC mice (arrows, MNs; scale bar = 50 μm). **(B)** Quantitative analysis of MNs in lumbar spinal cord of the two groups. **(C)** Representative images of MNs apoptosis which were determined by TUNEL staining. Scale bar, 50 μm. **(D)** Comparison of TUNEL staining in the MNs of the Tg-NC mice and the Tg-Optn SOD1^G93A^ mice at 90 days of age. **(E)** Western blot of Bcl2 and Bax expression in lumbar spinal cord of the Tg-Optn and Tg-NC mice. **(F,G)** Graph showing the quantitative analysis of Bcl2 and Bax. **(H)** Western blot of Cyt C expression in lumbar spinal cord of the Tg-Optn and Tg-NC mice. **(I)** Graph showing the quantitative analysis of Cyt C. *P < 0.05, **P < 0.01, and ***P < 0.001, *n* = 3 in each group.

As we know, an important pathological feature of ALS is the degeneration of MNs (Pasinelli and Brown, 2006). Thus, by counting large lumbar MNs within the anterior horn lamina IX using Nissl staining at 90 days of age, we found that MNs increased by 46.8% (*P* < 0.01) in the Tg-Optn group (575 ± 43) compared to the Tg-NC group (358 ± 63) in the L4-5 segments of SOD1^G93A^ mice (Figures 3A,B).

Apoptosis has been implicated as the main mechanism of neuronal death in ALS. Therefore, we then examined the effect of Optn overexpression on apoptosis by TUNEL staining. Our data reveled a significant decrease in the number of TUNEL-positive cells in the Tg-Optn group compared to the Tg-NC group (Figures 3C,D, *P* < 0.001). Additionally, Optn overexpression was found to regulate the expression of apoptosis-related factors Bcl-2 and Bax, resulting in a 30% decrease of the pro-apoptotic protein Bax and a 20% increase in the anti-apoptotic protein Bcl-2 (Figures 3E–G). Moreover, we found that cytochrome C (Cyt C) protein expression levels were significantly reduced in lumbar spinal cord of SOD1^G93A^ mice after Optn overexpression in the pre-symptomatic phase (Figures 3H,I).

### 3.4 Optn overexpression could have an anti-inflammatory effect in the pre-symptomatic phase

**FIGURE 4:**
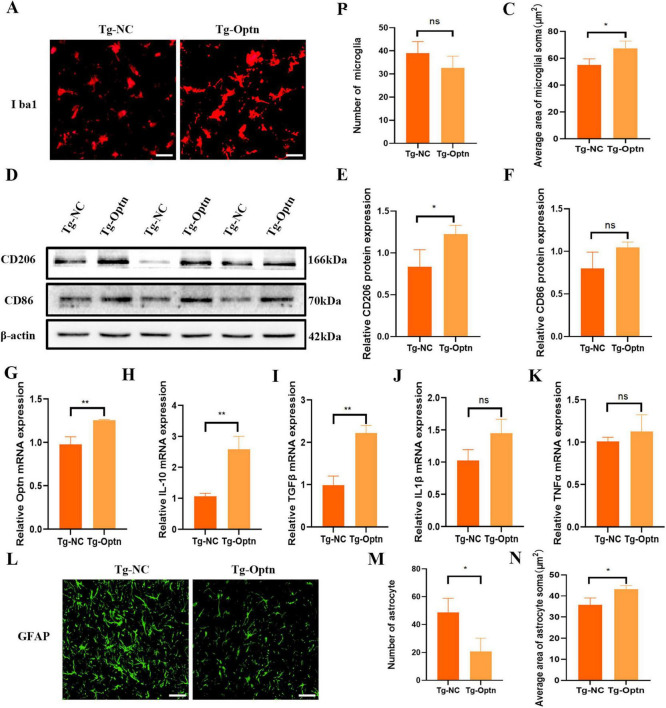
Optn overexpression regulated neuroinflammation in pre-symptomatic SOD1^G93A^ mice. **(A)** Immunofluorescence staining of Iba1 in lumbar spinal cord of the Tg-Optn and Tg-NC mice, scale bar = 50 μm. **(B)** Number of Iba1^+^ cells. **(C)** Average area of microglial soma in lumbar spinal cord. **(D)** Western blot of CD206 and CD86 expression. **(E,F)** Quantitative analysis of CD206 and CD86 protein level. **(G–K)** Quantitative analysis of Optn, IL-10, TGF-β, IL-1β, and TNF-α mRNA expression level. **(L)** Immunofluorescence staining of GFAP in lumbar spinal cord of the Tg-Optn and Tg-NC mice, scale bar = 50 μm. **(M)** Number of GFAP cells. **(N)** Average area of astrocytes soma in lumbar spinal cord. *n* = 3 in each group, *P < 0.05, **P < 0.01, and ns *P* > 0.05.

The inflammation of the CNS is a critical feature of ALS neuropathology, and NF-κB plays a cardinal role in cellular inflammatory and immune response processes. In our experiments, no statistically significant difference was achieved in the number of microglia (Figures 4A,B). However, Optn overexpression resulted in microglial activation as evidenced by larger microglial cytoplasm and shorter synapses compared to the Tg-NC group (Figures 4A,C). Conventionally, microglial activation could be distinguished into an anti-inflammatory M2 phenotype expressing CD206 and a pro-inflammatory M1 phenotype expressing CD86 (Kwon and Koh, 2020). The Western Blot results of our present study indicated a significant increase in CD206 expression and a non-significant trend of higher levels of CD86 (Figures 4D–F). Meanwhile, we observed that Optn overexpression led to a significant increase in the mRNA levels of the anti-inflammatory cytokines IL-10 and TGF-β in the pre-symptomatic phase. Conversely, there was no statistically significant change in the mRNA levels of the pro-inflammatory cytokines IL-1β and TNF-αin the pre-symptomatic phase. This suggests that Optn overexpression may selectively enhance anti-inflammatory pathways without obviously affecting pro-inflammatory cytokine expression between Tg-NC and Tg-Optn groups (Figures 4G–K). Moreover, there was a significant reduction in the astrocytes counts in the Tg-Optn group (Figures 4L,M). Meanwhile, the average area of astrocyte soma increased significantly in the Tg-Optn group (Figure 4N). These findings suggest Optn overexpression might have an anti-inflammatory effect in the pre-symptomatic phase.

### 3.5 Optn overexpression improved mitochondrial quality in MNs of the lumbar spinal cord in SOD1^G93A^ mice

**FIGURE 5:**
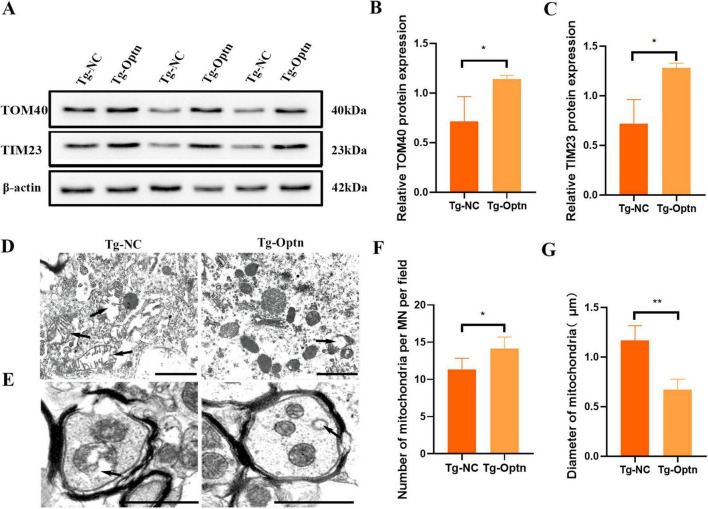
Optn overexpression improved mitochondrial quality. **(A)** Western blot analysis of TOM40 and TIM23. **(B,C)** Quantification of TOM40 and TIM23 protein, *n* = 3 in each group. **(D,E)** TEM images of mitochondria in the soma and axon of spinal MNs. the arrows point to giant and swollen mitochondria, scale bar = 1 μm. **(F,G)** Quantitative analysis of the number and Maximum diameter of mitochondria in the MNs of two groups, *n* = 5 in each group, *P < 0.05 and **P < 0.01.

Mitochondria, being vital organelles, plays a crucial role in providing cells with the energy necessary for life activities (Duann and Lin, 2017). These abnormalities encompass structural deformities, pathological swelling, excessive fission, dysregulated mitophagy, and a compromised mitochondrial membrane potential, ultimately disrupting cellular bioenergetics and neuronal homeostasis (Guo et al., 2024). Transporting proteins across the inner and outer mitochondrial membranes is crucial for maintaining mitochondrial function. This transportation process is facilitated by a series of protein complexes, including TOM complex in the outer membrane and the TIM23 complex in the inner membrane (Wiedemann and Pfanner, 2017). TOM and TIM are membrane protein complexes, with Tom40 serving as the core channel component of the TOM complex and Tim23 fulfilling a similar role in the TIM complex. Variations in their expression levels affect mitochondrial quality. Mitochondrial dysfunction plays a critical role in the onset and progression of ALS, and previous research, including TEM studies, has linked SOD1 mutations to a cascade of mitochondrial abnormalities. Specifically, in VSC4.1 cells expressing the SOD1^G93A^ mutation, increased mitochondrial ROS production leads to a decline in mitochondrial membrane potential, further impairing crucial mitochondrial functions such as the operational integrity of the TIM23 complex. Here, we observed an approximately 1-fold increase in the levels of the outer membrane transporter protein TOM40 and the inner membrane transporter protein TIM23 following Optn overexpression (Figures 5A–C). In addition, Optn overexpression was associated with fewer mitochondria undergoing swollen vacuolization in the MN soma and axon, according to TEM analysis (Figures 5D,E). Additionally, the anterior horn MNs in mice from the Tg-Optn group showed that the number of normal mitochondria increased by approximately 16.1% (11.33 ± 1.52 vs. 14.17 ± 1.52, *P* < 0.05) compared to those in the Tg-NC group (Figure 5F), while more giant and swollen mitochondria were found in the MNs soma of the Tg-NC group, and the average maximum diameter of the mitochondria decreased by approximately 41% in the Tg-Optn group (1.17 ± 0.15 μm vs. 0.67 ± 0.11 μm, *P* < 0.05) (Figure 5G). All these results indicated that Optn overexpression could improve mitochondrial quality in SOD1^G93A^ mice.

### 3.6 Optn overexpression regulates mitochondrial dynamics and promote mitophagy in SOD1^G93A^ mice

**FIGURE 6:**
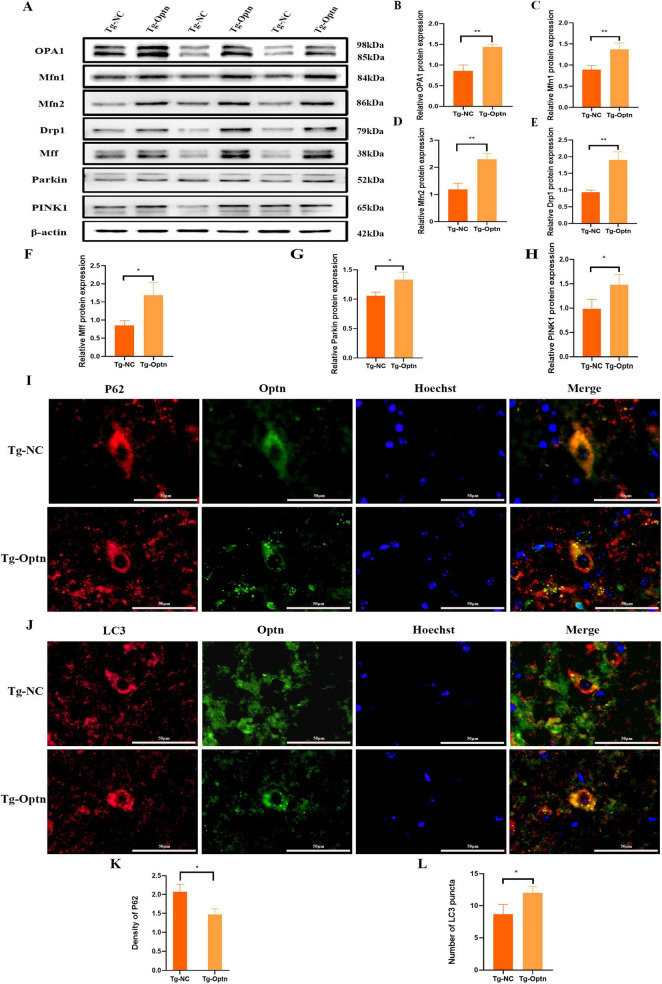
Optn overexpression enhanced mitophagy in SOD1^G93A^mice. **(A)** Western blot analysis of OPA1, Mfn1, Mfn2, Drp1, Mff, Parkin, and PINK1 expression. **(B–H)** Quantitative analysis of OPA1, Mfn1, Mfn2, Drp1, Mff, Parkin, and PINK1 protein level, *n* = 3 in each group. **(I)** Double immunostaining of P62 and Optn, scale bar = 50 μm. **(J)** Double immunostaining of LC3 and Optn, scale bar = 50 μm. **(K)** Quantitative analysis of P62 density. **(L)** Quantitative analysis of LC3 puncta, *n* = 3 in each group. *n* = 3 in each group. *P < 0.05 and **P < 0.01.

The maintenance of mitochondrial health and function is achieved through the mitochondrial quality control system including mitochondrial dynamics and mitophagy. Mitochondrial fusion and mitochondrial fission are the primary pathway to regulate the mitochondria morphology and mitochondrial dynamics. Previous studies have demonstrated that SOD1 mutations lead to reduced expression of mitochondrial fusion-associated proteins, specifically optic atrophic protein 1 (OPA1), Mitofusin 1 (Mfn1), and Mitofusin 2 (Mfn2) (Ferri et al., 2010). Furthermore, these mutations impaired autophagic flux, resulting in compromised degradation and subsequent intracellular accumulation of P62 (Zhang et al., 2014). Our data showed that Optn overexpression resulted in a significant up-regulation of mitochondrial fusion-associated proteins in SOD1^G93A^ mice, i.e., OPA1, Mfn1, and Mfn2 (Figures 6A–D). Meanwhile, the expression of mitochondrial division-related proteins Dynamin-related protein 1 (Drp1) and Mitochondrial fission factor (Mff) were also significantly elevated (Figures 6A,E,F), These results suggest that Optn may regulate mitochondrial dynamics by controlling fusion and division.

Mitophagy, the selective autophagy of dysfunctional mitochondria from the cytoplasm, is another important pathway for maintaining mitochondrial quality (Heinemeyer et al., 2019; Akabane et al., 2023). The PINK1/Parkin signaling is a key pathway in mitophagy regulation, in response to mitochondrial damage. PINK1 can phosphorylate and recruit Parkin to initiate autophagy via autophagy receptors such as OPTN (Lazarou et al., 2015). In the present study, we observed a significant increase in the protein expression levels of PINK1 and Parkin in the Tg-Optn group compared to the Tg-NC group (Figures 6A,G,H). Furthermore, the double immunostaining for LC3, P62, and Optn illustrated a marked increase in LC3 puncta and a decrease in P62 density in the Optn-positive cells of the Tg-Optn group in contrast to the Tg-NC group (Figures 6I–L). These findings indicate that Optn has the potential to stimulate mitophagy in SOD1^G93A^ mice.

## 4 Discussion

OPTN mutations are important cause of fALS, and several studies have shown that OPTN aggregation or loss is involved in the progression of ALS due to other gene mutations (Hortobágyi et al., 2011; Tak et al., 2020). OPTN is highly conserved and plays a crucial role in mitophagy and neuroinflammation (Wagner et al., 2008; Shen et al., 2015). Here, we observed a significant Optn increase in the spinal cord of the SOD1^G93A^ mice model of ALS at the pre-symptomatic stage, but a significant decrease after disease onset. Moreover, Optn overexpression could delay the disease onset and protect MNs through inhibiting cellular apoptosis, improving mitochondrial quality and regulating neuroinflammation in the pre-symptomatic phase of SOD1^G93A^ mice.

Our study reported the dynamics of Optn in the lumbar spinal cord of SOD1^G93A^ mice before and after disease onset (Figure 1), further supporting the idea that OPTN deficiency plays an important role in the apoptosis of MNs in ALS (Zhao et al., 2023). The upregulation of Optn expression may be a compensatory response to the pre-existing mitochondrial damage in the MNs of SOD1^G93A^ mice during the early stages of disease progression. It was plausible that Optn overexpression improved the impaired mitochondrial quality in MNs. Moreover, since Optn is predominantly expressed in neurons, the progression of the disease in SOD1^G93A^ mice appeared to exacerbate mitochondrial damage, leading to excessive Optn depletion over time and contributing to the decreased level in the lumbar spinal cord. OPTN dysfunction is thought to play an important role in the loss of MNs in ALS. To investigate this, we overexpressed Optn in symptomatic SOD1^G93A^ mice. However, this intervention did not prevent the disease onset of ALS. We hypothesized that the Optn overexpression might not be sufficient to protect impaired MNs after disease onset, overexpression of Optn genes was essential to exert a protective effect on MNs and mitochondria in the pre-symptomatic phase of SOD1^G93A^ mice. Indeed, Optn overexpression significantly delayed the onset of the disease and protected MNs in pre-symptomatic SOD1^G93A^ mice, which was also reported by Wen et al. The researchers found that Optn gene therapy was effective in increasing autophagy and protecting mitochondria to prevent progression in SOD1^G93A^ mice by delivering AAV9-Optn via intraspinal injection (Wen et al., 2024).

Our findings revealed that the Optn overexpression was associated with a reduction in MNs loss. The number of surviving MNs increased dramatically following treatment with Optn overexpression in the anterior horn of the lumbar spinal cord in SOD1^G93A^ mice, suggesting that motor function was effectively protected in the spinal cord of SOD1^G93A^ mice. Simultaneously, it was observed that the number of apoptotic cells had a notable reduction, highlighting the Optn overexpression-induced inhibition of abnormal cellular apoptosis by modulating the expression of Bcl-2 family proteins and cyt C (Figure 3). This finding is crucial as it suggests the potential to delay the onset of disease in SOD1^G93A^ mice. It is worth emphasizing that Optn overexpression also significantly enhanced locomotor activity of SOD1^G93A^ mice in the presymptomatic stage (Figure 2). Similarly, it is more vital to ensure the quality of life for ALS patients with mutations in the SOD1 gene. These positive outcomes prompt us to investigate the underlying mechanisms by which Optn overexpression exerts its beneficial roles in ALS progression.

The survival of MNs is highly dependent on the health and integrity of the mitochondria, which are responsible for the production of ATP and energy. Proper mitochondrial function relies on the smooth transport of mitochondrial proteins. Issues in the mitochondrial transport system can result in various complex human diseases, including cancers, neurodegenerative disorders, and muscular tissue-related disorders (Sorice et al., 2023). TOM40 and TIM23 act as transporters in the outer and inner mitochondrial membranes, respectively, and their increased expression levels can be used as an indicator of improved mitochondrial inner and outer membrane function (Araiso et al., 2019). In this study, we found that TOM40 and TIM23 were up-regulated after Optn overexpression, suggesting that mitochondrial inner and outer membrane transport functions were improved (Figure 5). Furthermore, TEM also confirmed Optn exerted a protection on mitochondrial morphology in MNs and axons. These results suggest that protecting mitochondrial function and regulating mitochondrial quality may be an important mechanism by which Optn overexpression attenuates MNs loss.

The maintenance of mitochondrial quality involves two interrelated processes: mitochondrial dynamics and mitophagy. Mitochondrial dynamics, characterized by continuous division and fusion, is vital for regulating the morphology, distribution, and quantity of mitochondria to maintain cellular homeostasis. Conversely, mitophagy plays an essential role in eliminating dysfunctional mitochondria. Interestingly, these two processes exhibit a reciprocal relationship, as they can mutually modulate each other to maintain the balance of mitochondrial quality (Wang et al., 2024). Under normal physiological conditions, mitochondria are able to maintain their quality through continuous fusion and fission. Mitochondrial fusion is induced by homotypic and heterotypic interactions between Mfn1, Mfn2 and OPA1 (Zhou et al., 2021). Mitochondrial fission is regulated by Mff and Drp1, whose deficiency accelerates MNs death and axonal degeneration in injured neurons (Kiryu-Seo et al., 2016). Mitophagy is another important pathway for maintaining mitochondrial quality (Wu et al., 2023). PINK1/Parkin-mediated mitophagy is the best-characterized machinery for clearing damaged mitochondria (Zhou et al., 2021). Previous studies have shown that the phenomenon of reduced expression of PINK1 and Parkin was found in models of ALS caused by SOD1 mutations (Knippenberg et al., 2013; Palomo et al., 2018). Our study demonstrated that Optn overexpression resulted in elevated expression levels of mitochondrial fusion-associated proteins OPA1, Mfn1, and Mfn2. This indicates that OPTN plays a role in removing damaged mitochondria and facilitating their fusion to avoid excessive mitochondrial aggregation by stimulating the production of mitochondrial fusion-associated proteins. Additionally, our results revealed an increase of mitochondrial fission-associated proteins Drp1 and Mff upon Optn overexpression, Optn maybe contribute to preventing the formation of enlarged mitochondria by participating in the mitochondrial fission pathway. Furthermore, we observed an increased level of mitophagy in SOD1^G93A^ mice overexpressing Optn, which was regulated by the expression of PINK1 and Parkin. Meanwhile, the results suggest that Optn overexpression leads to decreased accumulation of P62 and increased expression of LC3 (Figure 6). Therefore, Optn overexpression enhances mitochondrial quality by regulating dynamics and increasing mitophagy within MNs.

Neuroinflammation, involving activation of microglia and astrocytes, is a key aspect of ALS, although its specific impact on the disease is still under debate. Previous studies have shown that abnormal neuroinflammatory responses are important causes of ALS pathogenesis (Ransohoff, 2016; Yu et al., 2022). In the pathophysiology of ALS, it had been observed that the phenotype and functional status of microglia exhibited significant variability as the disease progresses. Several studies have proved that microglia show M2 phenotype and protect MNs at the onset of the disease, while microglia shift to M1 phenotype and aggravate the injury of MNs in ALS mice at terminal stage of the disease (Liao et al., 2012; Gravel et al., 2016; Du et al., 2020). This shift is accompanied by the release of various pro-inflammatory factors from microglia, which in turn triggers a neuroinflammatory response. Consequently, this neuroinflammatory state contributes to neuronal damage and eventual MNs death in ALS. Astrocytes show a toxic phenotype in ALS, which results in damage to MNs (Di Giorgio et al., 2007; Beers and Appel, 2019). In the SOD1 mutant ALS mice model and ALS patients at autopsy, significant astrocytes activation was detected near upper and lower MNs as well as in corticospinal tracts. The activated astrocytes exhibited elevated GFAP immunoreactivity (Vahsen et al., 2021; Gomes et al., 2022). The absence of Optn promoted NF-κB translocation to the nucleus and a large number of pro-inflammatory genes expression (Catanese et al., 2019; Harding et al., 2023). While, our study revealed that Optn overexpression promoted M2-type microglia activation, enhanced CD206 expression in M2 microglia, and suppressed GFAP activation in astrocytes. Furthermore, our findings indicate that Optn gene therapy markedly elevates the expression of anti-inflammatory factors such as IL-10 and TGF-β. These results indicate that Optn overexpression could regulate neuroinflammation in the spinal cord of SOD1^G93A^ mice.

This study may have some limitations. Firstly, we conducted analyses with a limited sample size of three participants. Future research would greatly benefit from a larger sample size to increase statistical power and strengthen the reliability of the findings. In addition, our study design did not adequately address the comparison of OPTN expression changes between these two periods after OPTN overexpression, which we recognize as a limitation of our approach. In our future research, we will conduct relevant studies to understand this issue in depth and thoroughly.

## 5 Conclusion

In conclusion, Optn dynamically changes before and after ALS onset in the lumbar spinal cord of SOD1^G93A^ mice. Overexpression of Optn could delay the onset of ALS and body weight loss by reducing MNs apoptosis, improving mitochondrial quality and regulating neuroinflammation. Therefore, it is worthwhile to investigate the effect of the Optn gene in other animal models of ALS.

## Data Availability

The original contributions presented in the study are included in the article/Supplementary material, further inquiries can be directed to the corresponding authors.

## References

[B1] Akabane S. Watanabe K. Kosako H. Yamashita S. I. Nishino K. Kato M. (2023). TIM23 facilitates PINK1 activation by safeguarding against OMA1-mediated degradation in damaged mitochondria. *Cell. Rep.* 42:112454. 10.1016/j.celrep.2023.112454 37160114

[B2] Araiso Y. Tsutsumi A. Qiu J. Imai K. Shiota T. Song J. (2019). Structure of the mitochondrial import gate reveals distinct preprotein paths. *Nature* 575 395–401. 10.1038/s41586-019-1680-7 31600774

[B3] Beers D. R. Appel S. H. (2019). Immune dysregulation in amyotrophic lateral sclerosis: Mechanisms and emerging therapies. *Lancet Neurol.* 18 211–220. 10.1016/s1474-4422(18)30394-6 30663610

[B4] Catanese A. Olde Heuvel F. Mulaw M. Demestre M. Higelin J. Barbi G. (2019). Retinoic acid worsens ATG10-dependent autophagy impairment in TBK1-mutant hiPSC-derived motoneurons through SQSTM1/p62 accumulation. *Autophagy* 15 1719–1737. 10.1080/15548627.2019.1589257 30939964 PMC6735587

[B5] Chen S. Zhang X. J. Li L. X. Wang Y. Zhong R. J. Le W. (2015). Histone deacetylase 6 delays motor neuron degeneration by ameliorating the autophagic flux defect in a transgenic mouse model of amyotrophic lateral sclerosis. *Neurosci. Bull.* 31 459–468. 10.1007/s12264-015-1539-3 26164555 PMC5563710

[B6] Di Giorgio F. P. Carrasco M. A. Siao M. C. Maniatis T. Eggan K. (2007). Non-cell autonomous effect of glia on motor neurons in an embryonic stem cell-based ALS model. *Nat. Neurosci.* 10 608–614. 10.1038/nn1885 17435754 PMC3139463

[B7] Du Y. Zhao W. Thonhoff J. R. Wang J. Wen S. Appel S. H. (2020). Increased activation ability of monocytes from ALS patients. *Exp. Neurol.* 328:113259. 10.1016/j.expneurol.2020.113259 32105709

[B8] Duann P. Lin P. H. (2017). Mitochondria damage and kidney disease. *Adv. Exp. Med. Biol.* 982 529–551. 10.1007/978-3-319-55330-6_27 28551805 PMC8049117

[B9] Feldman E. L. Goutman S. A. Petri S. Mazzini L. Savelieff M. G. Shaw P. J. (2022). Amyotrophic lateral sclerosis. *Lancet* 400 1363–1380. 10.1016/s0140-6736(22)01272-7 36116464 PMC10089700

[B10] Ferri A. Fiorenzo P. Nencini M. Cozzolino M. Pesaresi M. G. Valle C. (2010). Glutaredoxin 2 prevents aggregation of mutant SOD1 in mitochondria and abolishes its toxicity. *Hum. Mol. Genet.* 19 4529–4542. 10.1093/hmg/ddq383 20829229 PMC3298854

[B11] Gomes C. Sequeira C. Likhite S. Dennys C. N. Kolb S. J. Shaw P. J. (2022). Neurotoxic astrocytes directly converted from sporadic and familial ALS patient fibroblasts reveal signature diversities and miR-146a theragnostic potential in specific subtypes. *Cells* 11:1186. 10.3390/cells11071186 35406750 PMC8997588

[B12] Gravel M. Béland L. C. Soucy G. Abdelhamid E. Rahimian R. Gravel C. (2016). IL-10 controls early microglial phenotypes and disease onset in ALS Caused by misfolded superoxide dismutase 1. *J. Neurosci.* 36 1031–1048. 10.1523/jneurosci.0854-15.2016 26791230 PMC6601999

[B13] Guo Y. Guan T. Yu Q. Sanghai N. Shafiq K. Li M. (2024). ALS-linked SOD1 mutations impair mitochondrial-derived vesicle formation and accelerate aging. *Redox Biol.* 69:102972. 10.1016/j.redox.2023.102972 38056310 PMC10746562

[B14] Harding O. Holzer E. Riley J. F. Martens S. Holzbaur E. L. F. (2023). Damaged mitochondria recruit the effector NEMO to activate NF-κB signaling. *Mol. Cell.* 83 3188–3204.e3187. 10.1016/j.molcel.2023.08.005 37683611 PMC10510730

[B15] Heinemeyer T. Stemmet M. Bardien S. Neethling A. (2019). Underappreciated roles of the translocase of the outer and inner mitochondrial membrane protein complexes in human disease. *DNA Cell Biol.* 38 23–40. 10.1089/dna.2018.4292 30481057

[B16] Hortobágyi T. Troakes C. Nishimura A. L. Vance C. van Swieten J. C. Seelaar H. (2011). Optineurin inclusions occur in a minority of TDP-43 positive ALS and FTLD-TDP cases and are rarely observed in other neurodegenerative disorders. *Acta Neuropathol.* 121 519–527. 10.1007/s00401-011-0813-3 21360076

[B17] Jaiswal M. K. (2019). Riluzole and edaravone: A tale of two amyotrophic lateral sclerosis drugs. *Med. Res. Rev.* 39 733–748. 10.1002/med.21528 30101496

[B18] Kiryu-Seo S. Tamada H. Kato Y. Yasuda K. Ishihara N. Nomura M. (2016). Mitochondrial fission is an acute and adaptive response in injured motor neurons. *Sci. Rep.* 6:28331. 10.1038/srep28331 27319806 PMC4913268

[B19] Knippenberg S. Sipos J. Thau-Habermann N. Körner S. Rath K. J. Dengler R. (2013). Altered expression of DJ-1 and PINK1 in sporadic ALS and in the SOD1(G93A) ALS mouse model. *J. Neuropathol. Exp. Neurol.* 72 1052–1061. 10.1097/nen.0000000000000004 24128678

[B20] Koh S.-H. Kim Y. Kim H. Y. Hwang S. Lee C. H. Kim S. H. (2007). Inhibition of glycogen synthase kinase-3 suppresses the onset of symptoms and disease progression of G93A-SOD1 mouse model of ALS. *Exp. Neurol.* 205 336–346. 10.1016/j.expneurol.2007.03.004 17433298

[B21] Korac J. Schaeffer V. Kovacevic I. Clement A. M. Jungblut B. Behl C. (2013). Ubiquitin-independent function of optineurin in autophagic clearance of protein aggregates. *J. Cell. Sci.* 126(Pt 2), 580–592. 10.1242/jcs.114926 23178947 PMC3654196

[B22] Kwon H. S. Koh S. H. (2020). Neuroinflammation in neurodegenerative disorders: The roles of microglia and astrocytes. *Transl. Neurodegener* 9:42. 10.1186/s40035-020-00221-2 33239064 PMC7689983

[B23] Lazarou M. Sliter D. A. Kane L. A. Sarraf S. A. Wang C. Burman J. L. (2015). The ubiquitin kinase PINK1 recruits autophagy receptors to induce mitophagy. *Nature* 524 309–314. 10.1038/nature14893 26266977 PMC5018156

[B24] Liao B. Zhao W. Beers D. R. Henkel J. S. Appel S. H. (2012). Transformation from a neuroprotective to a neurotoxic microglial phenotype in a mouse model of ALS. *Exp. Neurol.* 237 147–152. 10.1016/j.expneurol.2012.06.011 22735487 PMC4126417

[B25] Maruyama H. Morino H. Ito H. Izumi Y. Kato H. Watanabe Y. (2010). Mutations of optineurin in amyotrophic lateral sclerosis. *Nature* 465 223–226. 10.1038/nature08971 20428114

[B26] Minegishi Y. Nakayama M. Iejima D. Kawase K. Iwata T. (2016). Significance of optineurin mutations in glaucoma and other diseases. *Progr. Retinal Eye Res.* 55 149–181. 10.1016/j.preteyeres.2016.08.002 27693724

[B27] Mori F. Tanji K. Toyoshima Y. Yoshida M. Kakita A. Takahashi H. (2012). Optineurin immunoreactivity in neuronal nuclear inclusions of polyglutamine diseases (Huntington’s, DRPLA, SCA2, SCA3) and intranuclear inclusion body disease. *Acta Neuropathol.* 123 747–749. 10.1007/s00401-012-0956-x 22318854

[B28] Nakamura M. Murray M. E. Lin W. L. Kusaka H. Dickson D. W. (2014). Optineurin immunoreactivity in neuronal and glial intranuclear inclusions in adult-onset neuronal intranuclear inclusion disease. *Am. J. Neurodegener. Dis.* 3 93–102. 25232514 PMC4162590

[B29] Palomo G. M. Granatiero V. Kawamata H. Konrad C. Kim M. Arreguin A. J. (2018). Parkin is a disease modifier in the mutant SOD1 mouse model of ALS. *EMBO Mol. Med.* 10:e8888. 10.15252/emmm.201808888 30126943 PMC6180298

[B30] Pasinelli P. Brown R. H. (2006). Molecular biology of amyotrophic lateral sclerosis: Insights from genetics. *Nat. Rev. Neurosci.* 7 710–723. 10.1038/nrn1971 16924260

[B31] Ransohoff R. M. (2016). How neuroinflammation contributes to neurodegeneration. *Science* 353 777–783. 10.1126/science.aag2590 27540165

[B32] Schneider C. A. Rasband W. S. Eliceiri K. W. (2012). NIH Image to ImageJ: 25 years of image analysis. *Nat. Methods* 9 671–675. 10.1038/nmeth.2089 22930834 PMC5554542

[B33] Shen W. C. Li H. Y. Chen G. C. Chern Y. Tu P. H. (2015). Mutations in the ubiquitin-binding domain of OPTN/optineurin interfere with autophagy-mediated degradation of misfolded proteins by a dominant-negative mechanism. *Autophagy* 11 685–700. 10.4161/auto.36098 25484089 PMC4502753

[B34] Sorice M. Profumo E. Capozzi A. Recalchi S. Riitano G. Di Veroli B. (2023). Oxidative stress as a regulatory checkpoint in the production of antiphospholipid autoantibodies: The protective role of NRF2 pathway. *Biomolecules* 13:1221. 10.3390/biom13081221 37627286 PMC10452087

[B35] Sundaramoorthy V. Walker A. K. Tan V. Fifita J. A. McCann E. P. Williams K. L. (2015). Defects in optineurin- and myosin VI-mediated cellular trafficking in amyotrophic lateral sclerosis. *Hum. Mol. Genet.* 24 3830–3846. 10.1093/hmg/ddv126 25859013

[B36] Tak Y. J. Park J. H. Rhim H. Kang S. (2020). ALS-related mutant SOD1 aggregates interfere with mitophagy by sequestering the autophagy receptor optineurin. *Int. J. Mol. Sci.* 21:7525. 10.3390/ijms21207525 33065963 PMC7590160

[B37] Vahsen B. F. Gray E. Thompson A. G. Ansorge O. Anthony D. C. Cowley S. A. (2021). Non-neuronal cells in amyotrophic lateral sclerosis - From pathogenesis to biomarkers. *Nat. Rev. Neurol.* 17 333–348. 10.1038/s41582-021-00487-8 33927394

[B38] Wagner S. Carpentier I. Rogov V. Kreike M. Ikeda F. Löhr F. (2008). Ubiquitin binding mediates the NF-kappaB inhibitory potential of ABIN proteins. *Oncogene* 27 3739–3745. 10.1038/sj.onc.1211042 18212736

[B39] Wang H. Luo W. Chen H. Cai Z. Xu G. (2024). Mitochondrial dynamics and mitochondrial autophagy: Molecular structure, orchestrating mechanism and related disorders. *Mitochondrion* 75:101847. 10.1016/j.mito.2024.101847 38246334

[B40] Wei Q. Zhou Q. Chen Y. Ou R. Cao B. Xu Y. (2017). Analysis of SOD1 mutations in a Chinese population with amyotrophic lateral sclerosis: A case-control study and literature review. *Sci. Rep.* 7:44606. 10.1038/srep44606 28291249 PMC5349524

[B41] Wen D. Ji Y. Li Y. Duan W. Wang Y. Li Z. (2024). OPTN gene therapy increases autophagy and protects mitochondria in SOD1-G93A-expressing transgenic mice and cells. *FEBS J.* 291 795–813. 10.1111/febs.17009 37983563

[B42] Wiedemann N. Pfanner N. (2017). Mitochondrial machineries for protein import and assembly. *Annu. Rev. Biochem.* 86 685–714. 10.1146/annurev-biochem-060815-014352 28301740

[B43] Wise J. P. Cannon J. (2016). From the cover: Alterations in optineurin expression and localization in pre-clinical parkinson’s disease models. *Toxicol. Sci.* 153 372–381. 10.1093/toxsci/kfw133 27473339 PMC5036620

[B44] Wu L. Wang L. Du Y. Zhang Y. Ren J. (2023). Mitochondrial quality control mechanisms as therapeutic targets in doxorubicin-induced cardiotoxicity. *Trends Pharmacol. Sci.* 44 34–49. 10.1016/j.tips.2022.10.003 36396497

[B45] Yu W. He J. Cai X. Yu Z. Zou Z. Fan D. (2022). Neuroimmune crosstalk between the peripheral and the central immune system in amyotrophic lateral sclerosis. *Front. Aging Neurosci.* 14:890958. 10.3389/fnagi.2022.890958 35592701 PMC9110796

[B46] Zhang J. J. Zhou Q. M. Chen S. Le W. D. (2018). Repurposing carbamazepine for the treatment of amyotrophic lateral sclerosis in SOD1-G93A mouse model. *CNS Neurosci. Ther.* 24 1163–1174. 10.1111/cns.12855 29656576 PMC6489874

[B47] Zhang X. Chen S. Song L. Tang Y. Shen Y. Jia L. (2014). MTOR-independent, autophagic enhancer trehalose prolongs motor neuron survival and ameliorates the autophagic flux defect in a mouse model of amyotrophic lateral sclerosis. *Autophagy* 10 588–602. 10.4161/auto.27710 24441414 PMC4091147

[B48] Zhao S. Chen R. Gao Y. Lu Y. Bai X. Zhang J. (2023). Fundamental roles of the Optineurin gene in the molecular pathology of Amyotrophic Lateral Sclerosis. *Front. Neurosci.* 17:1319706. 10.3389/fnins.2023.1319706 38178841 PMC10764443

[B49] Zhou H. Ren J. Toan S. Mui D. (2021). Role of mitochondrial quality surveillance in myocardial infarction: From bench to bedside. *Ageing Res. Rev.* 66:101250. 10.1016/j.arr.2020.101250 33388396

