# Correction: Optineurin overexpression ameliorates neurodegeneration through regulating neuroinflammation and mitochondrial quality in a murine model of amyotrophic lateral sclerosis

**DOI:** 10.3389/fnagi.2025.1670545

**Published:** 2025-08-05

**Authors:** Shumin Zhao, Ranran Chen, Yi An, Yali Zhang, Cheng Ma, Ying Gao, Yanchao Lu, Fei Yang, Xue Bai, Jingjing Zhang

**Affiliations:** ^1^Department of Neurology, Chifeng Municipal Hospital, Chifeng, China; ^2^Chifeng Clinical Medical College of Inner Mongolia Medical University, Chifeng, China; ^3^Medical Research Center, Chifeng Municipal Hospital, Chifeng, China; ^4^Intensive Care Unit, Chifeng Municipal Hospital, Chifeng, China

**Keywords:** amyotrophic lateral sclerosis, apoptosis, mitochondrial quality, neuroinflammation, optineurin

In the published article, there was an error in the **Funding** statement. The grant number for the funding source “Natural Science Foundation of Inner Mongolia Autonomous Region” was incorrectly listed as the application code (2022SHZR1527) when it should have instead been (2022MS08048). The corrected Funding statement and code appears below.

