# Supplementary material for: Optineurin overexpression ameliorates neurodegeneration through regulating neuroinflammation and mitochondrial quality in a murine model of amyotrophic lateral sclerosis
Source: Front Aging Neurosci. 2025 Feb 7;17:1522073. doi: 10.3389/fnagi.2025.1522073 (PMC11842329; doi:10.3389/fnagi.2025.1522073)
Supplement: Supplementary file 1 [file Data_Sheet_1.docx]

**Supplementary information**


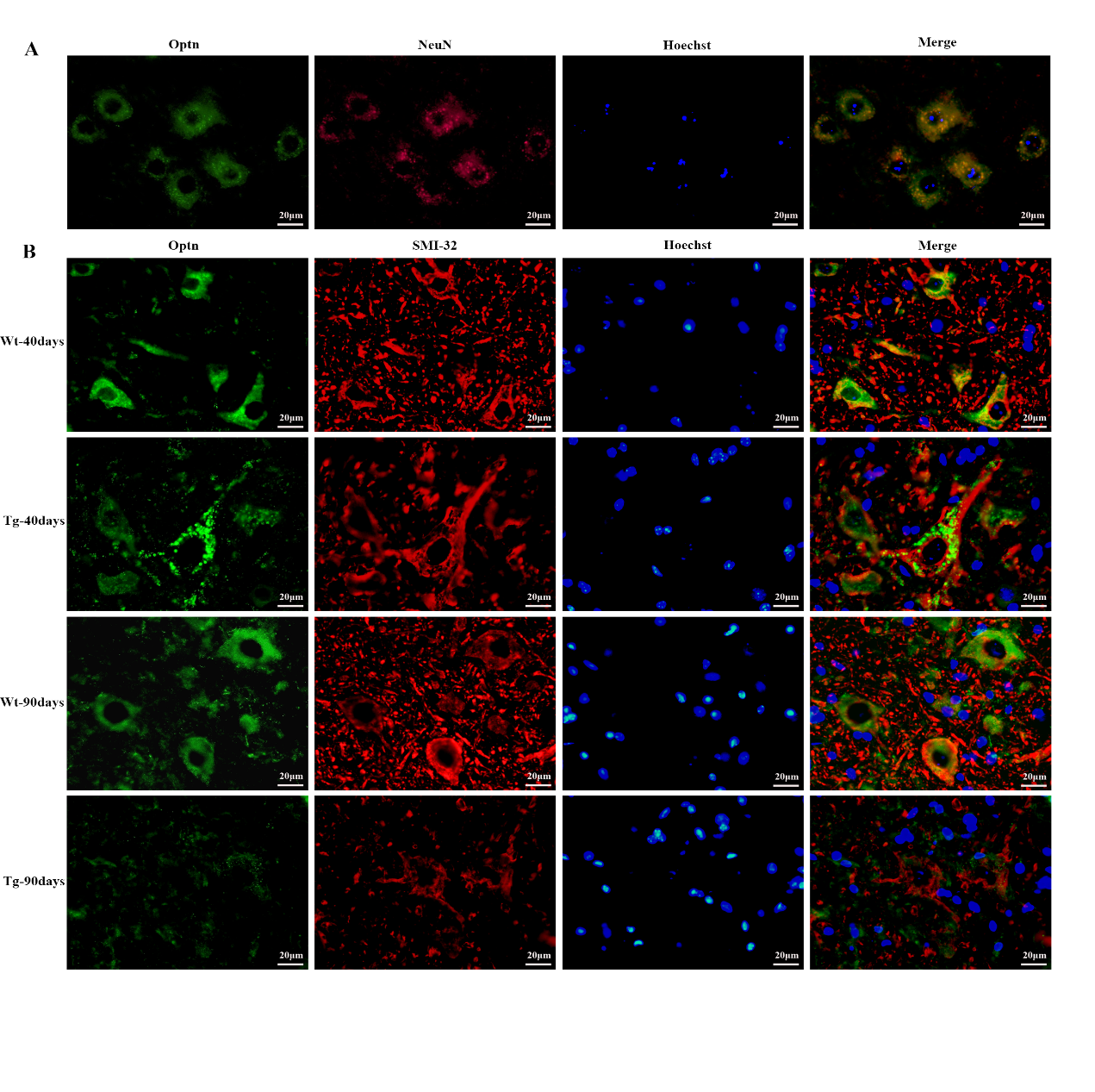


**Supplement figure 1.** Optn proteins are expressed on neurons. (A) Double immunostaining of Optn (green) and NeuN (red). Scale Bar=20μm. (B) Double immunostaining of Optn (green) and SMI-32 (red). Scale Bar=20μm.


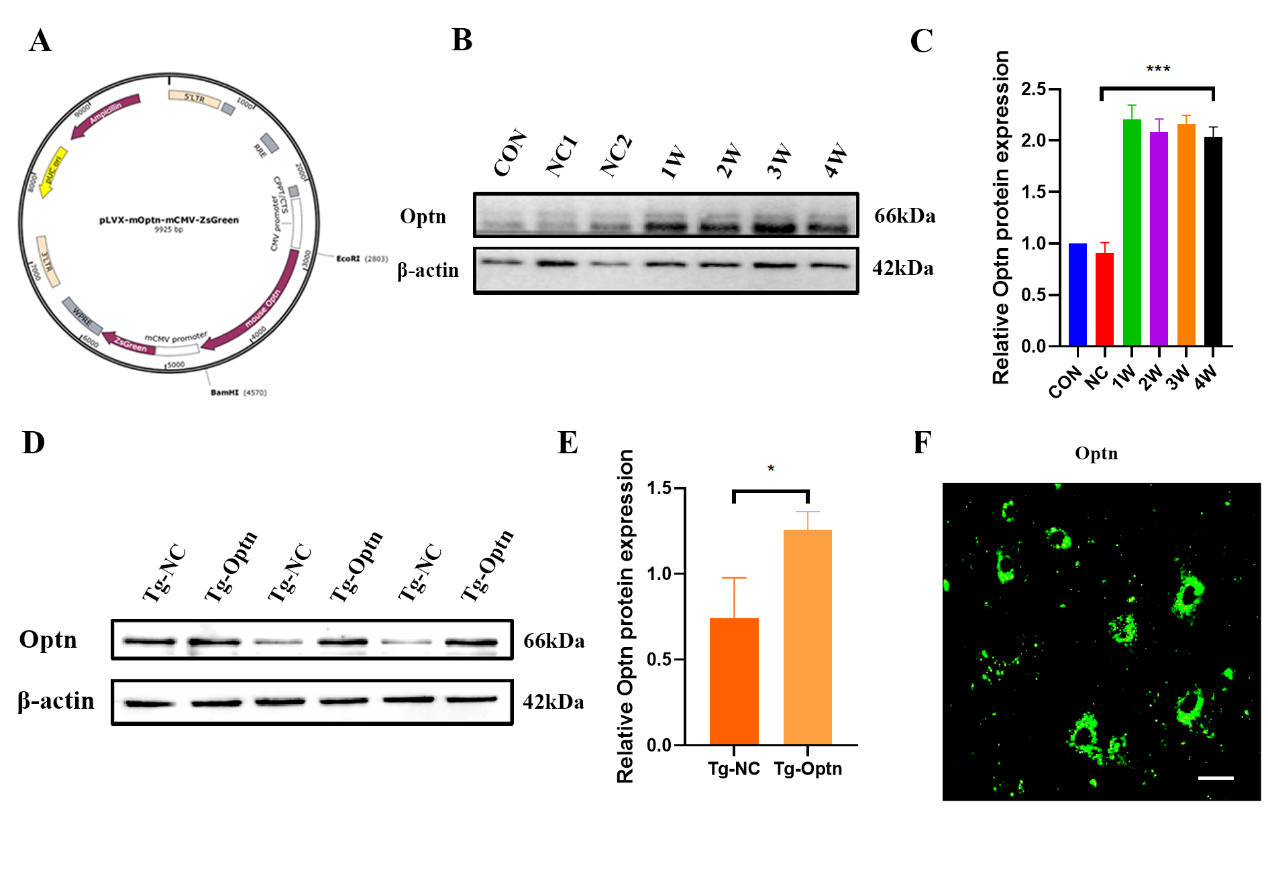


**Supplement figure 2.** Exogenous upregulation of Optn expression in Wt and Tg mice after injection of Optn lentivirus. (A) Schematic diagram of the Optn plasmids. (B) Immunoblots of Optn expression in the spinal cord of Tg mice at 1, 2, 3, and 4 weeks after lentivirus injection (n=3). (C) Analysis of Optn /actin at 1, 2, 3, and 4 weeks after lentivirus injection (n = 3). (D) Immunoblots of Optn expression in the spinal cord of Tg mice at 1 month after lentivirus injection. (E) Analysis of Optn /actin at 1 month after lentivirus injection. n = 4. (F) Fluorescent image of spinal cord sections from Tg mice 4 weeks after injection of Optn lentivirus (****P*＜0.001; **P*＜0.05; scale bar, 20 μm).


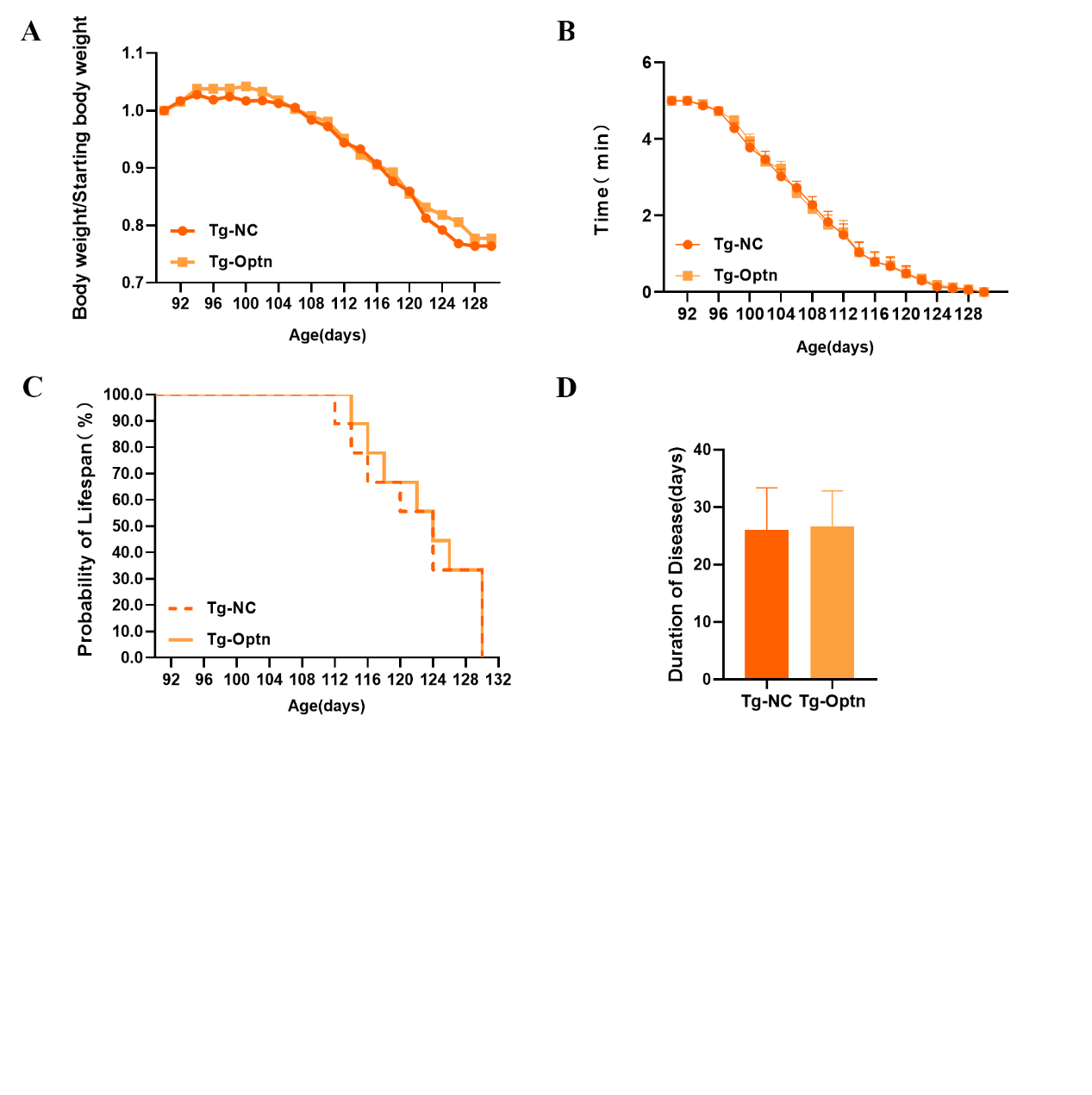


**Supplement figure 3.** Effects of Optn overexpression on lifespan and body weight of SOD1^G93A^ mice at the age of 90 days. (A) Body weight curves in the 2 groups. (B) Rotarod test at constant speeds of 20 rpm. (C) Kaplan-Meier survival analysis showing the probability of survival in Tg-NC and Tg-Optn mice. (D) Duration of Tg-NC and Tg-Optn mice. (n=9 in each group).
